# Bridging the Health Literacy Gap for Patients With Oral Cancer: Readability Enhancement With AI Chatbots

**DOI:** 10.1111/jop.70158

**Published:** 2026-06-11

**Authors:** Agata Baczynska, Umar Rehman, Shireen S. Gohari, Kelly Chu, Mohammad Sohaib Sarwar, Peter A. Brennan

**Affiliations:** ^1^ Imperial College London London UK; ^2^ UCL Division of Surgery and Interventional Science UCL London UK; ^3^ Department of Otolaryngology and Head and Neck Surgery St George's University Hospital NHS Foundation Trust London UK; ^4^ Department of Plastic Surgery, Lister Hospital East & North Hertfordshire NHS Trust Stevenage UK; ^5^ Department of Oral and Maxillofacial Surgery Bradford Royal Infirmary Bradford West Yorkshire UK; ^6^ Department of Oral and Maxillofacial Surgery Queen Alexandra Hospital Portsmouth UK

**Keywords:** content fidelity, health literacy, large language models, medical education, National Health Service (UK), oral cancer, patient information, readability

## Abstract

**Background:**

Early diagnosis is crucial in improving oral cancer outcomes. Patient education materials support timely recognition and management. However, these resources are often written above recommended reading levels, beyond patients' health literacy and limiting accessibility.

**Objectives:**

To assess the readability of available patient information on oral cancer by the NHS, to evaluate three large language models (LLMs; ChatGPT, Claude and Gemini) in simplifying texts while preserving their content, and to propose an improved leaflet based on UK materials, expert review and LLM adjustment to match average UK reading levels.

**Methods:**

Materials were collected from NHS‐affiliated websites. Original and LLM‐simplified texts were assessed using validated readability tools (FRES, FKGL, GFI, CLI and SMOG). Content fidelity was assessed using character 3–5‐g cosine, sentence‐content retention and latent semantic analysis (LSA). An expert review was applied to the proposed leaflet.

**Results:**

LLM‐revisions significantly improved readability across all five indices (*p* < 0.0001). Mean FRES of original texts was 66.4 ± 7.7, while Claude (81.6 ± 6.2) was the only model to surpass the 80 benchmark. Semantic similarity to source text remained high (LSA means 0.97 ± 0.04, 0.94 ± 0.09 and 0.96 ± 0.08; character 3–5‐g cosine 0.85 ± 0.05, 0.80 ± 0.08 and 0.82 ± 0.08 for respective models). Baseline readability of the proposed leaflet was comparable to NHS materials (FRES 65.7); Claude increased this to 81.2.

**Conclusions:**

LLM‐based simplification enhanced readability while preserving content fidelity. This approach can help enhance accessibility, particularly for populations disproportionately affected by oral cancer. With human oversight, it could be adopted at the policy level to standardise patient education and reduce health literacy disparities.

## 1 Introduction

### 1.1 Background

Oral cancer, most frequently squamous cell carcinoma (SCC), is a growing global health concern, with incidence predicted to rise by 65% in 2050 [1]. The disease and its treatments are associated with significant morbidity and mortality. It includes cancers of the lips (mucosal aspect), tongue, buccal mucosa, floor of the mouth, palate, alveolus (mandible and maxilla) and oropharynx. Major risk factors include tobacco use, excessive alcohol consumption, human papillomavirus (HPV) infection and areca (betel) nut chewing [2]. Notably, the incidence of oral cancer is disproportionately higher among individuals from socioeconomically disadvantaged backgrounds, who are also more likely to engage in high‐risk behaviours and present at advanced stages of disease [3].

Patient education plays a critical role in early detection and improved outcomes in oral cancer [4]. However, many patient information leaflets and online resources can be at a level that exceeds the average reading ability of the general public [5]. This mismatch in reading level, referred to as a health literacy gap, is known to limit understanding, reduce engagement and negatively affect outcomes [6]. Populations vulnerable to oral cancer may already face barriers to healthcare access, and this issue can significantly influence both prevention and early detection. Head and neck malignancies demonstrate similar socio‐economic disparities, with lower socio‐economic groups experiencing higher incidence rates and worse clinical outcomes [7].

Health literacy—defined as a person's ability to access, understand and use health‐related information—varies widely and is often lowest among individuals with lower education levels and incomes. In the United Kingdom, approximately 43% of working‐age adults are estimated to have literacy skills at or below those expected of an 11‐year‐old [8]. National guidelines recommend the patient‐facing materials be written at a fifth‐ to sixth‐grade reading level (approximately 11 years old) [8]. Despite these recommendations, many resources remain well above the recommended level [9, 10]. National Health Service (NHS) Long Term Plan [11] aspires to tackle health inequalities and expand digital patient information. Improving the readability of oral‐cancer materials is therefore a policy‐relevant priority.

Recent advances in large language models (LLMs) may offer a potential solution to this challenge. Generative AI models can rapidly adapt content to lower reading levels, offering a promising tool for bridging the health literacy gap [12, 13, 14]. However, a key concern is whether the simplified text accurately retains the original medical content. While prior studies have demonstrated improved readability, fewer have rigorously assessed content fidelity or incorporated expert clinical review [15, 16]. Objective approaches such as latent semantic analysis (LSA) provide a way to quantify whether the simplified text conveys the same overall meaning as the source material [17]. This is essential for ensuring that information for patients is not lost during simplification.

### 1.2 Study Objectives

By combining readability metrics and semantic comparison, the current study sought to explore the feasibility of using LLMs to make oral cancer education accessible regardless of educational status. In addition to evaluating materials provided by the NHS, a plain‐language oral cancer awareness leaflet was developed based on available material, expert review and LLM input. This was prompted by the scarcity of accessible resources.

## 2 Methods

### 2.1 Study Design and Setting

This study was conducted and reported in accordance with the CHART (Chatbot Health Advice Reporting Template) guideline for chatbot health advice studies [18]. The websites of all UK NHS Hospital Trusts, Health boards and Health and Social Care Trusts were searched for internet‐based patient education materials related to oral cancer between July and August 2025. Only written patient‐oriented materials, rather than articles describing interventions in oral cancer detection or videos, were taken into consideration.

### 2.2 Eligibility Criteria

Written, patient‐oriented online materials on oral cancer were included (e.g., leaflets, web pages for adult patients) with the requirement that they were produced by, or formally affiliated with, the NHS. Excluded formats were videos, purely graphical assets (e.g., posters/infographics without accompanying text), scholarly/professional articles and protocols describing interventions.

### 2.3 Readability Assessment

In line with established protocols, and to ensure accuracy of the readability score, copyright notices, adverts, author names, disclaimers, reference lists and all extraneous punctuation marks except periods were removed before readability assessment [9, 19]. Readability was assessed with Readable software [20] using five validated readability tools: Flesch Reading Ease Score (FRES), Flesch–Kincaid Grade Level (FKGL), Gunning Fog Index (GFI), Coleman–Liau Index (CLI) and Simple Measure of Gobbledygook Index (SMOG). Table S1 provides the interpretation of each tool and target scores used for this study. In line with the NHS Health Literacy Toolkit recommendation, the target score for FRES was a score of ≥ 80, and for FKGL, GFI, CLI and SMOG was a score of ≤ 6.9, consistent with the literacy level of an 11‐year‐old.

### 2.4 LLM‐Rewrites Generation

Following the initial readability assessment, each patient information leaflet was processed through three widely used public‐facing LLMs: ChatGPT‐5, Gemini 2.5 Pro and Claude Sonnet 4. Each model was prompted with the instruction: ‘Revise the provided patient education material to have a readability level comprehensible by an individual with the literacy level of an 11‐year‐old, while maintaining a tone suitable for an adult audience’. This prompt aligns with approaches used in prior studies exploring the use of generative AI for simplifying patient education materials [9, 10, 19].

### 2.5 Content Fidelity Assessment

To assess content fidelity between originals and LLM rewrites, we applied three complementary similarity measures. At the document level, we computed cosine similarity using L2‐normalised character 3–5‐g bag‐of‐n‐grams (consecutive word) representations, which provide a robust measure of overall textual similarity and highlight major lexical shifts. At the sentence level, recall, precision and harmonic mean (F1) were estimated by matching original to rewritten sentences using local‐window cosine similarity (threshold ≥ 0.70); this detects whether specific information was omitted (recall) or added/hallucinated (precision). Finally, semantic overlap was assessed via LSA, in which a TF–IDF term–document matrix (unigrams and bigrams) was reduced with singular value decomposition and cosine similarity computed in the latent space, enabling detection of preserved meaning even under extensive paraphrasing.

### 2.6 Development of the Proposed Leaflet

A plain‐language oral cancer patient information leaflet was developed by the research team as a proof‐of‐concept template to evaluate the potential role of LLM‐assisted refinement in improving patient‐facing written information. The intention of this work was not to produce a document for direct clinical implementation, but rather to explore methodological approaches for enhancing readability and structure of patient education materials.

The leaflet was adapted from the United Kingdom Plastics Research Collaborative (UKPRC) Patient Education Programme framework. As part of this study, publicly available oral cancer patient information leaflets had been undertaken and this was used as a basis to identify consistently reported and prioritised informational domains.

This synthesis informed the core content structure of the leaflet, which included definitions, risk factors, signs and symptoms and prevention. These domains were selected as they represented the most frequently and consistently included elements across patient‐facing resources and therefore provided a content‐valid foundation for leaflet development.

The initial leaflet was drafted without the use of AI tools. The drafting process involved synthesis of the identified domains into a coherent, plain‐language format suitable for an adult patient audience. Treatment‐related content was limited to safety‐netting guidance and advice on when to seek medical attention, rather than detailed descriptions of treatment modalities, consistent with standard patient information leaflet design.

Face and content validity of the draft leaflet were subsequently assessed through expert review by two consultant oral and maxillofacial/head and neck surgeons with specialist expertise in oral oncology. The leaflet (Supporting Information Appendix A) was refined based on this review of core themes of available leaflets. Readability was assessed using standardised readability metrics (Table S1).

Following completion of the human‐authored leaflet, AI‐generated rewrites were produced for comparative evaluation. These versions were not used in the initial drafting or content creation process but were generated post hoc to assess the impact of LLM‐assisted refinement on readability and content fidelity. This ensured appropriate readability metrics for patient‐facing material.

Two independent reviewers assessed each version against three criteria: (1) clinical accuracy of content, (2) fidelity to the intended message of the original leaflet and (3) suitability for an adult patient population. Responses were recorded as binary (‘yes’ or ‘no’) outcomes. This approach aligns with established methods for expert‐based content validity assessment in patient education research [21, 22].

### 2.7 Statistical Analysis

Each NHS Trust contributed paired observations across models (Original, ChatGPT, Claude and Gemini). Normally distributed readability metrics were compared using paired *t*‐tests with mean differences (Δ), 95% confidence intervals and Cohen's *dz* reported as effect sizes. Similarity metrics were compared using Friedman tests (non‐parametric repeated measures), with Kendall's *W* reported as effect size. Where omnibus results were significant, pairwise Wilcoxon signed‐rank tests with Holm correction for multiple comparisons were applied. Effect sizes are reported as Cohen's *dz* for parametric tests and rank‐biserial *r* for non‐parametric tests. Results are summarised as mean ± standard deviation and median [interquartile range]. Correlations between document‐level and sentence‐level similarity measures were estimated with Spearman's *ρ*, with Holm adjustment across tests. All analyses were conducted in R (version 4.3.2).

## 3 Results

### 3.1 Descriptive Data: Availability of Online Patient Information

Among regional NHS organisations providing an Otorhinolaryngology or Oral and Maxillofacial Surgery (OMFS) service across the United Kingdom, only 17/147 (11.6%) had any written oral‐cancer materials available online. The total of 26 identified materials came from different organisational levels: NHS England Trusts (14/26), NHS Cancer Alliances (2/26), NHS England General Practice (1/26), NHS Official Website (1/26), NHS‐affiliates Council resource (1/26), NHS Scotland Boards (3/26), NHS Health Scotland/NHS Inform resources (2/26), NHS Wales Official Website (1/26) and NHS Health Boards (1/26).

### 3.2 Readability

**FIGURE 1:**
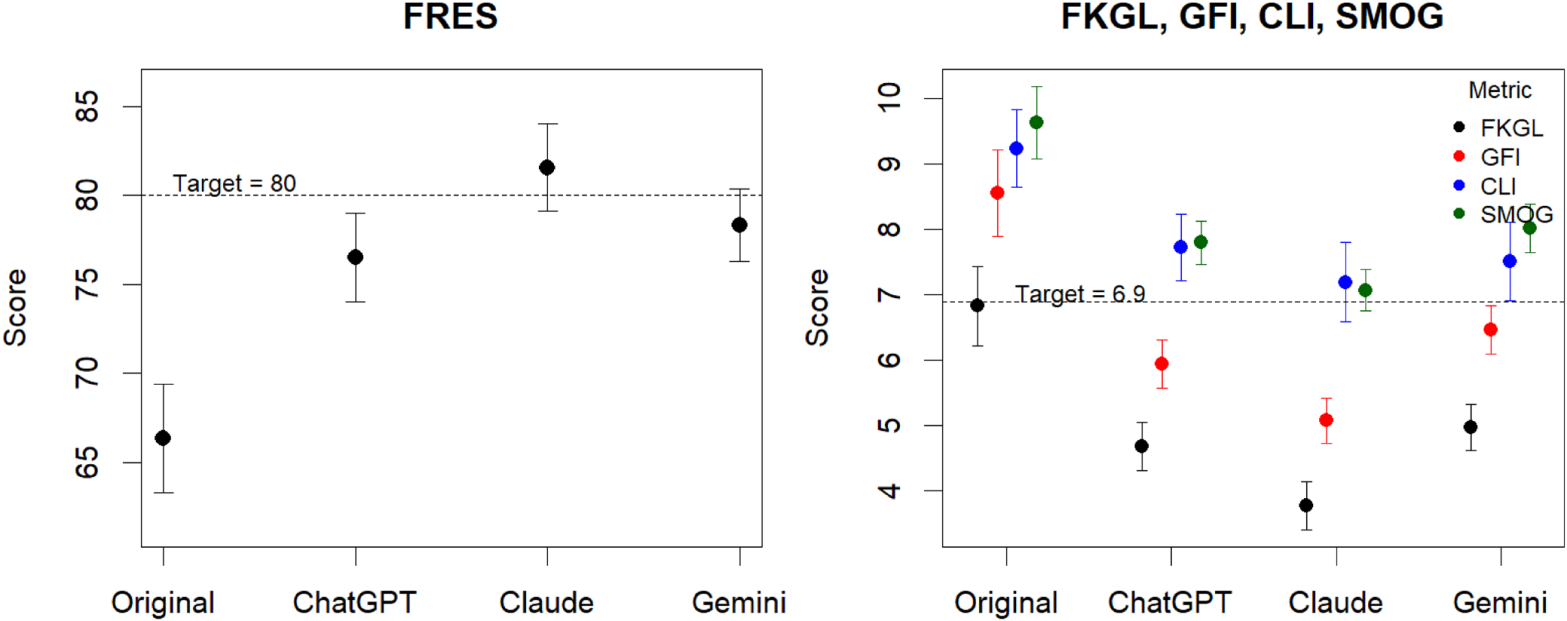
Readability of original NHS oral‐cancer materials compared with LLM rewrites. Left: Flesch Reading Ease Score (FRES; higher scores indicate greater readability, target ≥ 80). Right: Grade‐level indices including Flesch–Kincaid Grade Level (FKGL), Gunning Fog Index (GFI), Coleman–Liau Index (CLI) and Simple Measure of Gobbledygook (SMOG), where lower scores indicate easier readability (target ≤ 6.9). Across 26 NHS Trusts, all LLM outputs significantly improved readability relative to originals (all *p* < 0.0001). Claude produced the greatest gains, achieving the only FRES above target (81.6 ± 6.2) and the lowest grade‐level scores. ChatGPT and Gemini also improved readability substantially but did not meet all thresholds.

Across NHS Trust websites, LLM rewrites were consistently easier to read than the original materials. Original texts scored poorly on FRES (66.4 ± 7.7, below the ≥ 80 target) and exceeded the ≤ 6.9 threshold across FKGL, SMOG, GFI and CLI. All LLMs produced significant improvements on every index (*p* < 0.0001), with Claude showing the strongest gains and the only model to surpass the FRES benchmark (81.6 ± 6.2). ChatGPT (76.5 ± 6.3) and Gemini (78.3 ± 5.1) improved FRES but did not meet the target. For FKGL, all LLM versions met the ≤ 6.9 threshold (Original 6.8 vs. Claude 3.8, ChatGPT 4.7 and Gemini 5.0). SMOG scores also improved, with Claude (7.1), ChatGPT (7.8) and Gemini (8.0) all above the 6.9 target but still markedly reduced relative to Original (9.6). Similar improvements were observed for GFI (Original 8.6 vs. Claude 5.1, ChatGPT 5.9, Gemini 6.5) and CLI (Original 9.2 vs. Claude 7.2, ChatGPT 7.7 and Gemini 7.5). Omnibus Friedman tests confirmed significant within‐trust differences across all models (all *p* < 0.0001, W = 0.55–0.85). Results are shown in Tables 1 and 2 and Figure 1.

**TABLE 1:** Descriptive statistics for readability scores of original NHS oral‐cancer materials and LLM‐generated rewrites (ChatGPT, Claude, Gemini) across indices: Coleman–Liau Index (CLI), Flesch–Kincaid Grade Level (FKGL), Flesch Reading Ease (FRES), Gunning Fog Index (GFI) and Simple Measure of Gobbledygook (SMOG).

Metric	Model	*n*	Mean	SD	Median	Q1	Q3	Mean ± SD	Median [IQR]
CLI	ChatGPT	26	7.72	1.25	7.80	6.80	8.47	7.72 ± 1.25	7.80 [6.80–8.47]
CLI	Claude	26	7.19	1.49	7.00	6.23	7.68	7.19 ± 1.49	7.00 [6.23–7.68]
CLI	Gemini2	26	7.52	1.49	7.20	6.60	8.30	7.52 ± 1.49	7.20 [6.60–8.30]
CLI	Original	26	9.23	1.46	8.70	8.27	10.50	9.23 ± 1.46	8.70 [8.27–10.50]
FKGL	ChatGPT	26	4.68	0.91	4.65	4.08	5.30	4.68 ± 0.91	4.65 [4.08–5.30]
FKGL	Claude	26	3.77	0.91	3.60	3.18	4.00	3.77 ± 0.91	3.60 [3.18–4.00]
FKGL	Gemini	26	4.97	0.88	5.05	4.32	5.57	4.97 ± 0.88	5.05 [4.32–5.57]
FKGL	Original	26	6.83	1.50	6.40	5.62	8.12	6.83 ± 1.50	6.40 [5.62–8.12]
FRES	ChatGPT	26	76.50	6.27	76.40	71.95	81.75	76.50 ± 6.27	76.40 [71.95–81.75]
FRES	Claude	26	81.57	6.25	81.80	78.70	86.25	81.57 ± 6.25	81.80 [78.70–86.25]
FRES	Gemini	26	78.33	5.07	78.20	74.55	82.85	78.33 ± 5.07	78.20 [74.55–82.85]
FRES	Original	26	66.36	7.74	67.60	58.70	72.15	66.36 ± 7.74	67.60 [58.70–72.15]
GFI	ChatGPT	26	5.94	0.92	5.85	5.43	6.47	5.94 ± 0.92	5.85 [5.43–6.47]
GFI	Claude	26	5.07	0.87	4.95	4.60	5.40	5.07 ± 0.87	4.95 [4.60–5.40]
GFI	Gemini	26	6.47	0.91	6.50	5.70	7.00	6.47 ± 0.91	6.50 [5.70–7.00]
GFI	Original	26	8.55	1.64	8.35	7.30	9.55	8.55 ± 1.64	8.35 [7.30–9.55]
SMOG	ChatGPT	26	7.80	0.82	7.75	7.32	8.38	7.80 ± 0.82	7.75 [7.32–8.38]
SMOG	Claude	26	7.07	0.78	6.85	6.55	7.40	7.07 ± 0.78	6.85 [6.55–7.40]
SMOG	Gemini	26	8.02	0.91	8.10	7.20	8.67	8.02 ± 0.91	8.10 [7.20–8.67]
SMOG	Original	26	9.63	1.38	9.35	8.50	10.50	9.63 ± 1.38	9.35 [8.50–10.50]

*Note:* Values are presented as mean ± standard deviation (SD) and median [interquartile range, IQR]. Claude produced the most accessible outputs across most metrics, including achieving the FRE ≥ 80 target.

**TABLE 2:** Paired *t*‐tests comparing original texts with LLM outputs (ChatGPT, Claude, Gemini) across readability indices: Coleman–Liau Index (CLI), Flesch–Kincaid Grade Level (FKGL), Flesch Reading Ease (FRES), Gunning Fog Index (GFI) and Simple Measure of Gobbledygook (SMOG).

Metric	*n*	Mean Δ	CI low	CI high	*p*	Cohen *dz* effect	Compare
CLI	26	−1.51	−1.90	−1.12	< 0.001	−1.55	Original vs. ChatGPT
CLI	26	−2.04	−2.49	−1.60	< 0.001	−1.85	Original vs. Claude
CLI	26	−1.72	−2.03	−1.41	< 0.001	−2.26	Original vs. Gemini
FKGL	26	−2.15	−2.60	−1.67	< 0.001	−1.91	Original vs. ChatGPT
FKGL	26	−3.05	−3.54	−2.57	< 0.001	−2.56	Original vs. Claude
FKGL	26	−1.85	−2.22	−1.49	< 0.001	−2.05	Original vs. Gemini
FRES	26	10.13	8.00	12.26	< 0.001	1.88	Original vs. ChatGPT
FRES	26	15.20	13.03	17.37	< 0.001	2.77	Original vs. Claude
FRES	26	11.97	10.37	13.56	< 0.001	2.97	Original vs. Gemini
GFI	26	−2.61	−3.11	−2.11	< 0.001	−2.09	Original vs. ChatGPT
GFI	26	−3.47	−3.98	−2.97	< 0.001	−2.78	Original vs. Claude
GFI	26	−2.08	−2.54	−1.62	< 0.001	−1.82	Original vs. Gemini
SMOG	26	−1.83	−2.21	−1.44	< 0.001	−1.92	Original vs. ChatGPT
SMOG	26	−2.56	−2.95	−2.17	< 0.001	−2.64	Original vs. Claude
SMOG	26	−1.60	−1.95	−1.26	< 0.001	−1.90	Original vs. Gemini

*Note:* Reported values include mean differences (Δ), 95% confidence intervals (CI), *p* values and effect sizes (Cohen's *dz*). All models significantly improved readability across every metric (all *p* < 0.001).

### 3.3 Content Fidelity

**FIGURE 2:**
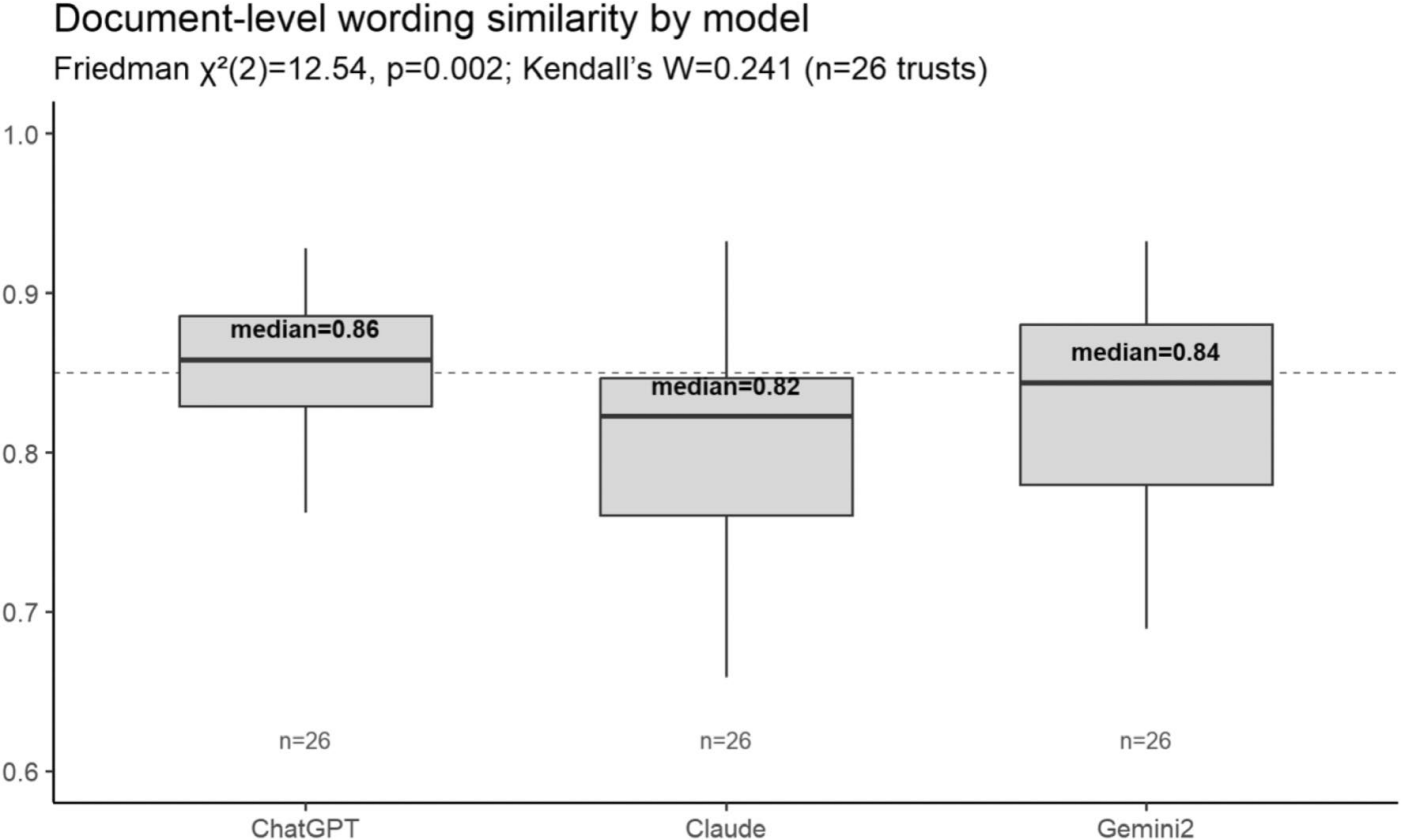
Document‐level wording similarity between original NHS oral‐cancer materials and LLM‐generated rewrites across 26 NHS Trusts. Cosine similarity was calculated using character 3–5 g. Median similarity scores were 0.86 (ChatGPT), 0.82 (Claude) and 0.84 (Gemini). A Friedman test indicated a small–moderate model effect (*χ* ^2^(2) = 12.54, *p* = 0.002; Kendall's *W* = 0.241). Post hoc Wilcoxon tests with Holm correction showed higher similarity for ChatGPT compared with Claude and Gemini, and for Gemini compared with Claude, though absolute differences were modest (~0.02–0.04 cosine units).

Across 26 leaflet sets, AI rewrites showed high surface‐form similarity to originals: character 3–5‐g cosine averaged 0.849 ± 0.054 (ChatGPT), 0.799 ± 0.078 (Claude) and 0.819 ± 0.083 (Gemini). A within‐trust Friedman test indicated a small–moderate model effect (*χ* ^2^(2) = 12.54, *p* = 0.002; Kendall's *W* = 0.241); post hoc Wilcoxon tests (Holm) showed ChatGPT > Claude, ChatGPT > Gemini and Gemini > Claude with modest absolute separations (~0.02–0.04 cosine units). Results are shown in Tables 3 and 4 and Figure 2.

**TABLE 3:** Descriptive statistics for semantic similarity metrics comparing original NHS texts with LLM rewrites.

Metric	Model	*n*	Mean	SD	CI_lo	CI_hi
Char35Cos (Original vs. AI)	ChatGPT	26	0.849	0.054	0.828	0.87
Char35Cos (Original vs. AI)	Claude	26	0.799	0.078	0.769	0.829
Char35Cos (Original vs. AI)	Gemini	26	0.819	0.083	0.787	0.851
F1_70 (sentence‐level)	ChatGPT	23	0.16	0.09	0.124	0.197
F1_70 (sentence‐level)	Claude	19	0.126	0.108	0.078	0.175
F1_70 (sentence‐level)	Gemini	19	0.144	0.199	0.055	0.233
Recall70 (sentence‐level)	ChatGPT	26	0.139	0.1	0.101	0.177
Recall70 (sentence‐level)	Claude	26	0.097	0.112	0.054	0.14
Recall70 (sentence‐level)	Gemini	26	0.126	0.194	0.052	0.201
Precision70 (sentence‐level)	ChatGPT	26	0.154	0.113	0.111	0.198
Precision70 (sentence‐level)	Claude	26	0.096	0.109	0.054	0.138
Precision70 (sentence‐level)	Gemini	26	0.099	0.177	0.031	0.167
LSA cosine (document‐level)	ChatGPT	26	0.972	0.044	0.955	0.989
LSA cosine (document‐level)	Claude	26	0.944	0.094	0.908	0.98
LSA cosine (document‐level)	Gemini	26	0.958	0.081	0.927	0.989

*Note:* Values are mean, standard deviation (SD) and 95% confidence intervals (CI).

**TABLE 4:** Pairwise post hoc comparisons for character 3–5‐g cosine similarity (Char35Cos).

Metric	Comparison	Median difference	Holm‐adjusted *p*	Effect size (*r*)
Char35Cos (Original vs. AI)	ChatGPT vs. Claude	0.0395	0.00165	0.677
Char35Cos (Original vs. AI)	ChatGPT vs. Gemini	0.0235	0.0271	0.433
Char35Cos (Original vs. AI)	Gemini vs. Claude	0.021	0.0199	0.505

At a strict 0.70 sentence‐match threshold, F1 was lower: 0.160 ± 0.090 (ChatGPT; *n* = 23), 0.126 ± 0.108 (Claude; *n* = 19), 0.144 ± 0.199 (Gemini; *n* = 19)—reflecting paraphrasing and sentence split or merge rather than content loss. Recall and precision were balanced. Using a more permissive threshold (0.65) increased F1 by +0.051, +0.059 and + 0.041, respectively, to 0.199, 0.167 and 0.165 (omnibus *χ* ^2^(2) = 7.28, *p* = 0.026; *W* = 0.192; pairwise ns).

In a single LSA space trained on all documents, Original ↔ AI cosine was near‐ceiling: 0.972 ± 0.044, 0.944 ± 0.094 and 0.958 ± 0.081, confirming preservation of clinical terminology and topics.

Taken together, AI rewrites largely retained content (high LSA, high document‐level similarity) while rephrasing sentences, which lowers strict sentence‐level *F*1 without indicating substantive information loss.

### 3.4 Proposed Leaflet

To address the scarcity of accessible oral cancer information, we developed a plain‐language awareness leaflet adapted from the UKPRC Patient Education Programme. The baseline leaflet (Supporting Information Appendix A), following expert review, scored FRES = 65.7, FKGL = 5.8, GFI = 7.0, CLI = 8.3 and SMOG = 8.6. Benchmarking against NHS summaries (Table 5) showed similar FRES to the NHS mean but lower grade‐level indices on all four measures, indicating simpler text even before LLM adjustment. Following LLM simplification, Claude produced the most readable version (FRES 81.2) (Supporting Information Appendix B) and was rated acceptable on expert review for clinical accuracy, fidelity to the source message and adult suitability. Detailed readability and fidelity metrics for all AI‐enhanced versions are reported in Table S2.

**TABLE 5:** Readability benchmarking of the proposed leaflet against NHS summary statistics.

Metric	NHS mean ± SD	Leaflet	Glass's Δ (*z*)	Percentile
FRES	66.4 ± 7.7	65.7	−0.09	46%
FKGL	6.8 ± 1.5	5.8	−0.67	25%
GFI	8.6 ± 1.6	7.0	−1.00	16%
CLI	9.2 ± 1.5	8.3	−0.60	27%
SMOG	9.6 ± 1.4	8.6	−0.71	24%

*Note:* Glass's Δ = (Leaflet − NHS mean)/SD; percentile is the position within the NHS distribution.

## 4 Discussion

### 4.1 Key Results

This study demonstrates that, when presented with a standardised prompt, LLMs can simplify patient education materials for oral cancer, while preserving the original meaning and semantic content. ChatGPT, Claude and Gemini have improved all readability indices when compared to the original text, with Claude surpassing the FRES threshold of ≥ 80. In addition to acceptable readability levels, surface‐level wording between original and AI‐generated texts remains largely similar, representing preserved content and virtually identical meaning, as evidenced by cosine similarity and near‐ceiling LSA values. Sentence‐level retention values were conversely lower for LLM‐rewrites, indicating a degree of sentence splitting, merging and paraphrasing, which was driving the increase in readability.

### 4.2 Interpretation

These findings are consistent with previously published studies applying LLMs to simplify patient‐facing medical information [9, 10, 15, 19]. Chatbots are successful in providing comprehensible yet accurate explanations of the following components commonly included in widely available patient materials: basic information, risk factors, symptoms, diagnosis and management. Our results extend the current literature by specifically validating LLM performance in oral cancer education, where complex medical terminology and multifaceted treatment approaches can create comprehension barriers for patients. This provides a simple yet powerful approach that is both cost‐effective and rapid to implement. If adopted at a policy level, LLM‐assisted simplification could standardise patient education across the country, reduce regional disparities and ensure that all patients receive information aligned with recommended health literacy levels. However, it should be noted that commercial LLM outputs are subject to model updates and retraining, meaning outputs may vary across users or time points; achieving true standardisation would therefore require additional safeguards including human oversight, prompt versioning and systematic output validation.

### 4.3 Implications for Practice and Policy

The potential applications for LLMs in patient education extend considerably beyond text simplification. Previous research has demonstrated that publicly available AI chatbots are highly effective in explaining oncology concepts to patients [23, 24, 25]. Studies have also explored the readability metrics for patient information generated de novo by LLMs [26, 27] and the empathetic qualities of AI responses, which, in some cases, are perceived by patients as more compassionate than in‐person clinical encounters [28]. Within oral cancer specifically, research remains more limited: one study examining the utility of AI chatbots as an interactive educational tool for oral cancer patients noted fragmented conversational flow despite high expert‐rated accuracy and usability [29], while another evaluated AI's responses to common oral cancer questions using a 5‐point empathy scale, with ChatGPT scoring highly [30].

Our approach offers a unique advantage by combining AI capabilities with traditional educational formats. By using LLMs to enhance written materials rather than requiring direct patient interaction with chatbots, this method addresses accessibility concerns for populations who may be less confident with newer technologies, including older adults and those from lower socioeconomic backgrounds [31]. Importantly, written resources such as leaflets not only serve patients with existing diagnoses but also play a vital role in prevention and early detection [32], providing information to individuals who may not yet recognise their risk or be aware of what questions to ask—particularly relevant in oral cancer, where risk is shaped by modifiable behaviours. In this respect, AI‐enhanced leaflets complement chatbot‐based tools, with each serving distinct but equally important functions in the patient‐education pathway.

Beyond enhancing existing materials, the broader potential of LLMs in cancer education is substantial. These tools could be leveraged to explain treatment options and their associated side effects in patient‐appropriate language, simplifying complex surgical, radiotherapy and chemotherapy protocols into understandable terms; however, it is important to underline the significance of clinical oversight to ensure accuracy and appropriateness. Furthermore, LLMs could guide patients in follow‐up care and self‐monitoring, providing clear instructions to recognise warning signs, manage treatment‐related symptoms and adhere to post‐treatment surveillance schedules when developed and validated by healthcare professionals. These systems could potentially offer further reassurance and evidence‐based clarifications to address common patient concerns and misconceptions.

### 4.4 Addressing Health Inequalities

The clinical applications are particularly significant for addressing health inequities. Readable patient‐facing materials can help reduce barriers to comprehension for patients with lower education or literacy levels, who disproportionately belong to lower socioeconomic backgrounds, older age groups and minority ethnic populations [33]—the same groups more likely to present late with oral cancer [3]. Such materials could be deployed across primary care, dental practices and community outreach programmes to target vulnerable populations, reinforcing the importance of designing materials not only for average reading levels but specifically for vulnerable subgroups. This is pertinent given the prevalence of misinformation in cancer care and the tendency of emotionally distressed patients to seek information from unreliable online sources [34, 35]. This approach supports NHS guidance on patient‐facing information [36] and directly aligns with the NHS Long Term Plan, which prioritises reducing health inequalities, strengthening prevention and earlier cancer diagnosis and expanding digital‐first patient information [11]. Utilising LLM‐assisted workflows within these settings could help standardise readable resources across Trusts, consider population diversity when creating educational materials and support delivery in resource‐limited or underserved areas. Notably, the present study was conducted exclusively using English‐language materials; validation of LLM‐assisted simplification in non‐English languages represents an important priority for future work.

### 4.5 Evidence Gaps and Resource Development

Our findings also highlight pertinent gaps in the current provision of oral cancer patient information. Of note, most NHS Trusts do not provide any form of online patient information pertaining to oral cancer. This absence represents a critical gap in accessible patient‐facing resources, particularly given the importance of early detection in oral cancer prognosis and the role of education in supporting timely recognition and management. In addition to evaluating existing NHS patient information, we developed a plain‐language oral‐cancer leaflet adapted from the UKPRC Patient Education Programme using an iterative process of human editing and LLM‐assisted simplification. The human‐edited version achieved readability scores similar to NHS materials, despite being designed to improve readability. Subsequent LLM simplification improved the indices, with Claude meeting the majority of the thresholds. Following expert clinical review, AI‐enhanced versions were deemed satisfactory resources for patient education and could be feasibly implemented across different NHS Trusts. This illustrates that combining targeted design with AI‐assisted drafting can generate patient information that is substantially more accessible and adaptable for other conditions. Future work developing patient information leaflets for clinical deployment should incorporate co‐development with patient and public involvement groups alongside clinical experts, with LLMs used as supportive tools for refinement rather than primary content generation. In addition, formal comparative studies assessing performance against existing NHS oral cancer patient information leaflets in real‐world clinical settings are required to establish external validity and patient‐centred effectiveness. However, these evaluations were beyond the scope of the present proof‐of‐concept study.

### 4.6 Strengths and Limitations

A key strength of this study is the focus on content fidelity in combination with readability assessment. Although previous studies have examined the readability improvements possible with LLM‐assisted simplification, our study supplements this assessment with an analysis of the content to ensure meaning is also preserved. The use of semantic and cosine similarity, and sentence‐content retention provides us with objective metrics to critically review the similarity of LLM‐revised texts to originals. This provides us with the unique perspective to demonstrate how sentence structure can be modified to improve accessibility while remaining near‐identical to the original text and retaining informational accuracy.

This study has some limitations. It focused exclusively on publicly available NHS‐affiliated materials, thereby omitting any which may not be publicly available and potentially overlooking other relevant patient information in the United Kingdom. Therefore, the study was limited by its relatively small number of patient information materials. While sufficient to demonstrate consistent effects, it limits generalisability; larger and more geographically diverse datasets are needed for future studies. Despite this, our use of reliable, objective measures and the consistency of our findings with prior literature strengthen confidence in the validity of the results.

A further limitation relates to the reproducibility of LLM outputs. As commercial models are continuously updated, identical prompts may yield different outputs over time and the goal of consistent patient education cannot be achieved through LLM simplification alone without appropriate governance and validation frameworks.

## 5 Conclusion

We have demonstrated that LLMs can effectively enhance the readability of oral cancer patient education materials while preserving semantic content. The ability of LLMs to achieve recommended readability thresholds offers a scalable, cost‐effective approach to addressing health literacy gaps that disproportionately affect vulnerable populations at the highest risk of oral cancer in line with the NHS Long Term Plan priorities. While human oversight remains essential, LLM‐assisted simplification provides a practical tool for standardising patient education, potentially reducing health inequities and improving patient engagement in oral cancer prevention and care.

## Conflicts of Interest

The authors declare no conflicts of interest.

## Supporting information


**Supporting Information: Appendix A.** Human‐authored plain language proposed leaflet.

**Supporting Information: Appendix B.** Claude‐enhanced version of the proposed leaflet.

**Table S2:** Combined readability, similarity/coverage and expert‐review metrics for the three AI‐enhanced UKPRC leaflets. Readability indices: higher FRES is better; lower FKGL, GFI, CLI, SMOG are better. Char35Cos = document‐level character 3–5‐g cosine versus original. Recall70/Precision70/F170 use sentence‐level 3–5‐g cosine with a 0.70 threshold and bi‐directional coverage. LSA values were computed on the global corpus. Expert‐review cells show the count of reviewers (out of 2) who agreed.

**Table S1:** Interpretation of readability scoring tools.

## Data Availability

The data that support the findings of this study are available in the Supporting Information of this article.
