# Supplementary material for: Bridging the Health Literacy Gap for Patients With Oral Cancer: Readability Enhancement With AI Chatbots
Source: J Oral Pathol Med. 2026 Jun 11;55(8):936–45. doi: 10.1111/jop.70158 (PMC13533865; doi:10.1111/jop.70158)
Supplement: Supplementary file 1 [file JOP-55-936-s001.docx]

**Supplementary Appendix A.** Human-authored plain language proposed leaflet.

# Introduction

Oral cancer accounts for 2% of all cancer diagnoses in the UK with 7500 new cases annually. It affects different parts of the mouth, including the tongue, gums, inner cheeks, lips, the roof and floor of the mouth.

Up to 46% of oral cancers can be prevented by avoiding risk factors and finding the growth early.

Timely detection allows for better chances of successful treatment and recovery.

# Risk factors

Some people are more likely to develop oral cancer due to a combination of lifestyle and health factors.

These include:

·	Tobacco use. Around 75% of people with oral cancer smoke. This includes cigarettes, cigars, pipes, and chewing tobacco.

·	Betel nut chewing. Common in some cultures, this mixture often contains tobacco, spices, lime and the areca betel nut.

·	Drinking alcohol. Especially in large amounts or combined with tobacco.

·	Poor diet. Not eating enough fresh fruits and vegetables can increase your risk.

·	Infections. Certain germs like Candida (a type of yeast) and some viruses may play a role.

·	Weakened immune system. People with reduced immunity are more vulnerable.

·	Sun exposure. For lip cancer too much sun without protection can be a contributor.

# Signs and symptoms

Look out for the following red flag signs.

·	A sore or ulcer in the mouth that doesn’t heal within 2 weeks.

·	White or red patches inside the mouth or on the tongue.

·	Unexplained bleeding or numbness.

·	A lump or thickening in the cheek or on the tongue.

·	Difficulty chewing, swallowing, or moving your jaw or tongue.

·	Persistent mouth pain or a feeling that something is stuck.

·	Changes in voice or speech.

·	Swelling or lumps in the neck.

# Diagnosis

If you experience any of these symptoms for more than 2-3 weeks, get checked.

Your dentist or doctor can examine your mouth and neck. They may refer you to a specialist.

# Prevention

You can reduce your risk of developing oral cancer by making healthy lifestyle choices.

·	Don’t smoke or chew tobacco.

·	Limit alcohol.

·	Eat a healthy diet.

·	Avoid betel nut chewing.

·	Visit your dentist regularly.

·	Protect your lips from the sun.

·	Talk to your doctor about HPV vaccination.
