# Supplementary material for: Bridging the Health Literacy Gap for Patients With Oral Cancer: Readability Enhancement With AI Chatbots
Source: J Oral Pathol Med. 2026 Jun 11;55(8):936–45. doi: 10.1111/jop.70158 (PMC13533865; doi:10.1111/jop.70158)
Supplement: Supplementary file 2 [file JOP-55-936-s002.docx]

**Supplemental Table 1.** Interpretation of readability scoring tools

| **Flesch Reading Ease Score** | | | | |
| --- | --- | --- | --- | --- |
|  | Score | Readability description | Approximate grade level | Target |
| FRES | 90-100 | Very easy | 5 | ≥80 |
|  | 80-90 | Easy | 6 |  |
|  | 70-80 | Fairly easy | 7 |  |
|  | 60-70 | Standard | 8-9 |  |
|  | 50-60 | Fairly difficult | 10-12 |  |
|  | 30-50 | Difficult | College |  |
|  | 0-30 | Very difficult | Graduate |  |
| **Grade-level based readability tools** | | | | |
| Tool | Interpretation | | | Target |
| FKGL | Years of education required to understand most of the text | | | ≤6.9 |
| GFI | Years of formal education a person requires to easily understand the text on first reading | | | ≤6.9 |
| CLI | Years of education required to understand most of the text | | | ≤6.9 |
| SMOG | Reading grade level necessary for a person to have reached in order to fully understand text | | | ≤6.9 |

FRES: Flesch Reading Ease, FKGL: Flesch-Kincaid Grade Level, GFI: Gunning Fog Index, CLI: Coleman-Liau Index, SMOG: Simple Measure of Gobbledygook.

**Note:** A target of ≤6.9 corresponds to the recommended readability level for public health materials (reading age of ~11 years / end of Key Stage 2 in UK education system).
