# Supplementary material for: Bridging the Health Literacy Gap for Patients With Oral Cancer: Readability Enhancement With AI Chatbots
Source: J Oral Pathol Med. 2026 Jun 11;55(8):936–45. doi: 10.1111/jop.70158 (PMC13533865; doi:10.1111/jop.70158)
Supplement: Supplementary file 3 [file JOP-55-936-s003.docx]

**Supplemental Table 2**. Combined readability, similarity/coverage, and expert-review metrics for the three AI-enhanced UKPRC leaflets. Readability indices: higher FRES is better; lower FKGL, GFI, CLI, SMOG are better. Char35Cos = document-level character 3–5-gram cosine vs Original. Recall70/Precision70/F170 use sentence-level 3–5-gram cosine with a 0.70 threshold and bi-directional coverage. LSA values were computed on the global corpus. Expert-review cells show the count of reviewers (out of 2) who agreed.

| **Model** | **FRES** | **FKGL** | **GFI** | **CLI** | **SMOG** | **Char35Cos** | **Recall70** | **Precision70** | **F170** | **LSA** | **Clinical accuracy (n/2)** | **Content fidelity (n/2)** | **Suitability (n/2)** |
| --- | --- | --- | --- | --- | --- | --- | --- | --- | --- | --- | --- | --- | --- |
| ChatGPT | 72.0 | 5.6 | 6.4 | 7.9 | 8.1 | 0.881 | 0.438 | 0.452 | 0.444 | 0.990 | 2 | 2 | 2 |
| Claude | **81.2** | 4.2 | 5.5 | 7.5 | 7.2 | 0.776 | 0.094 | 0.120 | 0.105 | 0.934 | 2 | 2 | 2 |
| Gemini | 77.9 | 4.9 | 6.4 | 7.2 | 8.0 | 0.732 | 0.062 | 0.065 | 0.063 | 0.934 | 2 | 2 | 2 |
