# Supplementary material for: Bridging the Health Literacy Gap for Patients With Oral Cancer: Readability Enhancement With AI Chatbots
Source: J Oral Pathol Med. 2026 Jun 11;55(8):936–45. doi: 10.1111/jop.70158 (PMC13533865; doi:10.1111/jop.70158)
Supplement: Supplementary file 4 [file JOP-55-936-s004.docx]

**Supplementary Appendix B.** Claude-enhanced version of the proposed leaflet.

**Oral Cancer: What You Need to Know**

**What is Oral Cancer?**

Oral cancer affects the mouth. It can grow on your tongue, gums, cheeks, lips, or the roof and floor of your mouth. In the UK, about 7,500 people get oral cancer each year. That's 2 out of every 100 cancer cases.

The good news? You can prevent almost half of all oral cancers by avoiding risks and catching them early. When found early, treatment works much better.

**Who Gets Oral Cancer?**

Some people have a higher chance of getting oral cancer. Here's what puts you at risk:

**Tobacco use** - This is the biggest risk. About 75 out of 100 people with oral cancer smoke. This includes cigarettes, cigars, pipes, and chewing tobacco.

**Betel nut chewing** - Some cultures chew a mix that has tobacco, spices, lime, and betel nuts.

**Drinking alcohol** - Heavy drinking raises your risk. Drinking and smoking together is even worse.

**Poor eating habits** - Not eating enough fruits and vegetables can increase your risk.

**Infections** - Some germs like yeast and certain viruses may cause problems.

**Weak immune system** - When your body can't fight illness well, you're at higher risk.

**Too much sun** - For lip cancer, sun damage without protection is a problem.

**Warning Signs to Watch For**

See your doctor or dentist right away if you have any of these problems for more than 2-3 weeks:

- A sore in your mouth that won't heal
- White or red spots in your mouth or on your tongue
- Bleeding in your mouth for no reason
- Numb spots in your mouth
- A lump in your cheek or on your tongue
- Hard time chewing, swallowing, or moving your jaw or tongue
- Mouth pain that won't go away
- Feeling like something is stuck in your mouth
- Your voice sounds different
- Lumps or swelling in your neck

Don't wait - get checked if these symptoms last more than 2-3 weeks.

**Getting Help**

Your dentist or doctor can check your mouth and neck. They will look for anything that seems wrong. If they find something, they may send you to a specialist for more tests.

**How to Protect Yourself**

You can lower your risk by making smart choices:

- **Don't smoke or chew tobacco** - This is the most important thing you can do
- **Drink less alcohol** - If you drink, keep it small amounts
- **Eat healthy foods** - Eat lots of fruits and vegetables
- **Don't chew betel nuts**
- **See your dentist regularly** - They can spot problems early
- **Protect your lips from the sun** - Use lip balm with sun protection
- **Ask about HPV shots** - Talk to your doctor about this vaccine

Remember: Finding oral cancer early gives you the best chance for successful treatment. Don't ignore warning signs, and make healthy choices to protect yourself.
